# Editorial: New trends in psychiatric research: Toward the clinical characterization of the individual case and the personalization of treatments

**DOI:** 10.3389/fpsyt.2022.1042536

**Published:** 2022-10-06

**Authors:** Gaia Sampogna, Peter Falkai, Tomasz Gondek, Andrea Fiorillo

**Affiliations:** ^1^Department of Psychiatry, University of Campania “L. Vanvitelli”, Naples, Italy; ^2^Department of Psychiatry and Psychotherapy, Ludwig-Maximilians-University (LMU) Munich University Hospital, Munich, Germany; ^3^Self-employed, Wrocław, Poland

**Keywords:** clinical characterization, dimensional, categorical approach, individual patient, personalization of treatment

Research in psychiatry is undergoing major changes due to continuous upheavals in psychological, social and environmental factors which have an impact on mental health and mental disorders (1). In particular, there is now an increasing interest toward personalized medicine in order to put the patient at the core of the care pathway (2). From a diagnostic viewpoint, it is recognized that many syndromes cannot be anymore diagnosed by simply fulfilling categorical criteria, but many symptoms overlap among psychiatric syndromes. Many of what we call mental disorders, although not qualifying at the moment as proper “disease entities,” are indeed patterns of observed signs and reported symptoms that trained clinicians have been able to recognize for decades in a variety of clinical contexts (3). Psychiatrists need a conceptual framework which acknowledges the complexity of mental disorders and indicates the type of information to be collected in order to personalize individualized treatment plans. There is the need to further characterize the individual patient with a given mental disorder, besides its signs and symptoms, in order to personalize the management plan and to promote patients' full personal recovery.

However, research evidence is still fragmentary, the selection of pharmacological and psychosocial treatments is usually based on clinicians' and/or patients' preferences as well as on safety issues, in a trial-and-error fashion, paying little or no attention to the particular features of the specific case. In ordinary clinical practice, psychiatrists refer to adopt a personalized approach for patients with severe mental disorders, although systematic guidelines are missing (4). This is why many patients suffering from severe mental disorders, such as major depression or schizophrenia, do not achieve remission with the first treatment they receive (5). Therefore, the more precise clinical characterization of these patients will enhance the provision of personalized management and the likelihood of obtaining optimal outcomes (6).

Orienting research toward the clinical characterization of the individual patient should lead to a renewed interest toward a transdiagnostic approach of mental disorders, rather than a categorical one (7). Indeed, the most recent editions of both ICD and DSM classification systems have tried to include some dimensional aspects—such as for the classification of personality disorders—confirming the need for a comprehensive and balanced evaluation of different psychopathological domains (8, 9).

The clinical characterization of the individual patient requires the evaluation of several domains, including clinical symptoms, severity of illness, clinical subtypes, level of functional status, staging of illness, medical and psychiatric comorbidities, early life adverse event(s), personality traits, environmental stressors, recovery styles and coping strategies (10–12). All these aspects are transdiagnostic and have been often overlooked by younger generation of psychiatrists, looking only at number of symptoms needed to fulfill diagnostic criteria. A transdiagnostic approach is useful also for the identification of risk and protective factors of mental disorders. As recently pointed out by Arango et al. (13), transdiagnostic risk/protective factors—such as childhood adversities, stressful events, being second generation immigrant—are mostly involved in the early neurodevelopmental period (14). The evidence is rapidly growing on the interaction between gene-by-environment-by-time of exposure, highlighting that environmental factors underly much of the variation in clinical and neurobiological phenotypes of mental disorders and their outcomes (15, 16).

New trends of research include the study of people in specific phases of their life (such as the perinatal period) (17–19) or in specific settings (such as refugee camps or war conflicts) (20, 21) in order to identify the impact of specific social stressors as possible risk factors for the development of mental disorders. Finally, although the neurobiological approach has failed to detect the “unique cause” of mental disorders, the progress in the identification and clinical use of biomarkers will be facilitated through multidimensional clinical assessment. It is indeed plausible that biomarkers will be found to correlate more closely with dimensions of psychopathology than with categorical diagnostic measures, which often hide important treatment-relevant aspects of illness. As such, biomarkers may become more useful as predictive factors, mediators or moderators of the variability in outcome response (22–24).

In the present Research Topic entitled “New trends in psychiatric research: Toward the clinical characterization of the individual case and the personalization of treatments” several original studies have addressed the issue of the personalization and individual characterization of each single patient from different point of view. In particular, some studies have been focused on the role of psychological resources and vulnerabilities as predictors of good outcome in different psychotherapies or on the sex-based differences in patients with affective disorders (Menculini et al.). Moreover, we have included papers dealing with the impact of the COVID-19 pandemic on patients' wellbeing and mental health, as well as on peripheral biomarkers of inflammation and its association with personal functioning (Chumakov et al.).

There is the need to further promote research in this field, but we hope that contributions collected in this Research Topic will be useful to improve the clinical characterization of the individual patients.

## Author contributions

All authors listed have made a substantial, direct, and intellectual contribution to the work and approved it for publication.

## Conflict of interest

The authors declare that the research was conducted in the absence of any commercial or financial relationships that could be construed as a potential conflict of interest.

## Publisher's note

All claims expressed in this article are solely those of the authors and do not necessarily represent those of their affiliated organizations, or those of the publisher, the editors and the reviewers. Any product that may be evaluated in this article, or claim that may be made by its manufacturer, is not guaranteed or endorsed by the publisher.
